# Quarterly Report of Proceedings

**Published:** 1857-01

**Authors:** 


### QUARTERLY REPORT OF THE PROCEEDINGS.

*Monday, Nov.* 11, 1856. The session for 1856-7 was opened, at the rooms, No. 37, Soho Square, by B. G. Babington, M.D., F.R.S., President of the Society. He congratulated the members on the adjustment of the eastern question, and the consequent restoration of peace to Europe. The President observed, that the whole of our army, and the greater part of the fleet, had either returned home, or had been dispersed over our various colonial stations; and that the naval and military Medical officers, the Sanitary Commissioners, and the medical men employed at the civil hospitals in the east, during the war, had now time to collect the results of their experience in a field of observation, only too extensive, both as regards the course of epidemic diseases, and the casualties incident to active, warlike operations.

"On the Mode of Investigating some Epidemic Diseases. By Gavin Milroy, M.D." In this paper the author made allusion to several important facts that had come under his observation, while serving as Sanitary Commissioner in the Crimea.

A discussion followed, in which Dr. Snow, Dr. Camps, Dr. Greenhow, Dr. Babington, and Dr. McWilliam took part.

*Monday, Dec.* 3, 1856. "A Sketch of the Principal Features of the Climate of the Crimea, and its effects upon Health, as observed during the first year of the occupation by the Allied Forces. By William Smart, M.D., H.M.S. *Diamond*, late Surgeon in charge of the Naval Brigade Hospital at Balaklava." Read by Dr. McWilliam.

Dr. Milroy, Mr. Rawlinson, Dr. Snow, Dr. Greenhow, and Dr. McWilliam discussed the paper; and Dr. Milroy and Mr. Rawlinson both highly eulogised Dr. Smart's conduct, which had come under their observation while in the Crimea, as being that of a most able and zealous medical officer.

